# Brain metastasis in a patient with melanoma receiving Pembrolizumab therapy: A case report and review of the literature

**DOI:** 10.1097/MD.0000000000009278

**Published:** 2017-12-15

**Authors:** Jin-cheng Song, Xiao-lei Ding, Xiu-hua Sun, Mohammed Safi, Juan Tian

**Affiliations:** Department of Oncology, the Second Affiliated Hospital of Dalian Medical University, Dalian, China.

**Keywords:** brain metastasis, melanoma, Pembrolizumab

## Abstract

**Rationale:**

Melanoma with brain metastasis is associated with a poor prognosis and high mortality rate. As patients with this condition have been excluded from most clinical trials, data on the use of anti–programmed death 1 therapy for these patients are limited.

**Patient concerns:**

The patient was a 62-year-old man with a 10-year history of melanotic nevus in his right forearm. He was admitted to another hospital in August 2015 due to the growth of the melanotic nevus over 1 year and complaint of a mass in the right mid-axillary area. The patient had no relevant medical, surgical, or family history.

**Diagnoses:**

The biopsy of his right axillary lymph node showed malignant melanoma.

**Interventions:**

He was subsequently treated with adjuvant high-dose interferon after dacarbazine. Numerous metastatic lesions were found in his lung, abdomen, pelvic cavity, and brain after five months later, and then Pembrolizumab was used for six cycles (2 mg/kg every 3 weeks). He experienced immunorelated adverse events and we gave him cortisol to treat immunorelated disease until pneumonia was found.

**Outcomes:**

We observed a delayed effect after three cycles of Pembrolizumab, the intracranial lesion presented clear margins and localization, while the other lesions became much smaller. A mixed response was observed after four cycles, with still stable extracranial metastases but growing a new lesion in brain. After two additional cycles of Pembrolizumab, the treatment was stopped due to the patient's inability to pay for it and a decline in his performance status. He then received palliative treatment at a local hospital and died for severe pulmonary infection, with an overall survival time of 7 months from metastasis.

**Lessons:**

In the case reported here, a delayed and mixed response was observed after Pembrolizumab was used. Because of causing severe pulmonary infection, the use of steroids should be considered carefully when treating immunorelated adverse events. It seemed that the Pembrolizumab has a positive effect on melanoma brain metastases especially combined with other treatments. However, there are still some challenges including patient selection, predictors of response, drug tolerance, optimizing combination strategies and control of adverse effects. More carefully designed clinical trials are urgently needed.

## 1 Introduction

Melanoma is the most aggressive skin cancer derived from melanocytes. An estimated 20,000 Chinese patients are newly diagnosed each year, with a 3–5% annual growth rate.^[1]^ The median overall survival (OS) time is <1 year, and 5-year survival rates of metastatic disease do not exceed 10%.^[2]^ Given the difficulty of early diagnosis, up to 37% of patients with metastatic melanoma have developed brain metastases, with a high mortality rate and median OS time of <6 months.^[3]^ Nevertheless, approximately 5% of patients with melanoma brain metastases (MBM) survive long term with standard management options, such as surgery, stereotactic radiation, whole-brain radiation therapy (WBRT), chemotherapy, targeted therapy, or combined treatment.^[4]^ In clinical studies, the cytotoxic T-lymphocyte-associated protein 4 (CTLA-4) inhibitor Ipilimumab has shown activity in patients with untreated MBM.^[5]^ The programmed death 1(PD-1) inhibitor Pembrolizumab appears to be more effective, with fewer adverse effects than the CTLA-4 inhibitor, but data on the use of anti-PD-1 therapies in patients with such metastasis are limited. Due to concerns about potential neurological sequelae of brain metastases, most clinical trials have excluded affected patients.^[6]^ Here, we describe a case of advanced melanoma with brain metastases that was treated with Pembrolizumab and review the literatures.

## 2 Case report

The patient was a 62-year-old man with a 10-year history of melanotic nevus in his right forearm. He was admitted to another hospital in August 2015 due to the growth of the melanotic nevus over 1 year and the complaint of a mass in the right mid-axillary area. The patient had no relevant medical, surgical, or family history. Ultrasound revealed an enlarged lymph node (4 × 4 cm) in the right mid-axillary area. The melanotic nevus was excised and the right axillary lymph node was dissected completely. The biopsy showed malignant melanoma with 1.5 mm invasion. No test for Raf murine sarcoma viral oncogene homolog B (BRAF V600E) mutation was conducted. The patient then presented to our department. Physical examination and ultrasound revealed enlarged lymph nodes in the right (2.5 × 0.6 cm) and left (2.7 × 0.5 cm) groin areas. Enhanced computed tomography (CT) and brain magnetic resonance imaging (MRI) showed no other lesion. In September 2015, the patient started 4 cycles of dacarbazine (500 mg/day for 5 consecutive days every 21 days). He was subsequently treated with adjuvant high-dose interferon (IFN), which was increased gradually from 3 to 12 million units.

**Figure 1:**
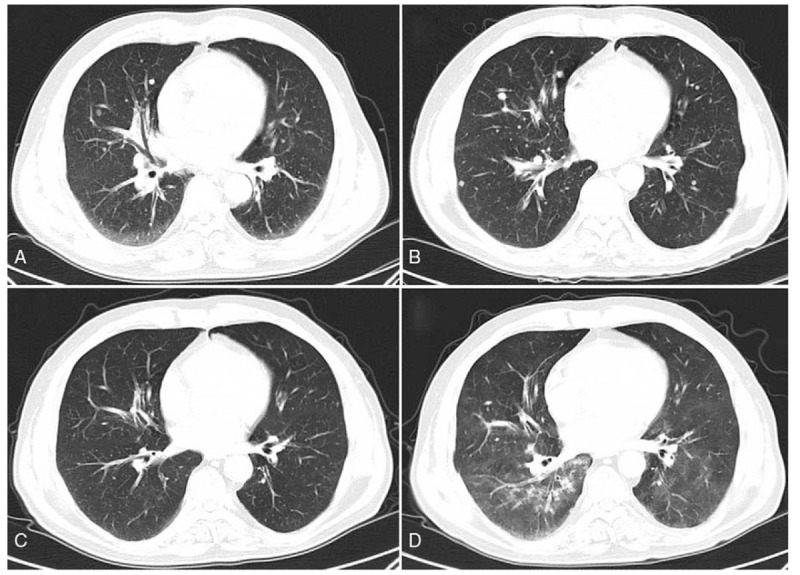
Enhanced CT images of the lungs before and after 2 to 4 cycles of pembrolizumab therapy. (A) Many lesions were present in both lobes before pembrolizumab treatment. (B) The lesions increased in number and size after 2 cycles of treatment. (C) The number and size of lesions in both lungs were significantly reduced after 3 cycles of treatment. (D) Interstitial pneumonia was found, especially in the right lobe, after 4 cycles of treatment.

**Figure 2:**
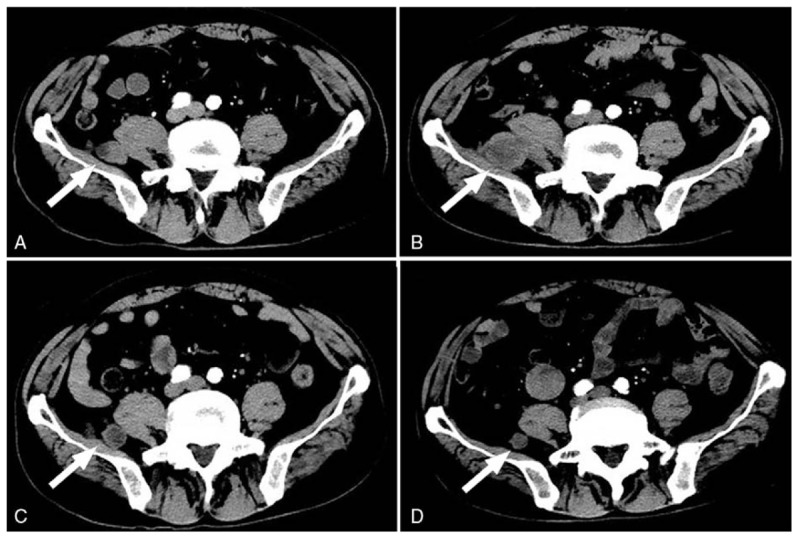
Enhanced CT images of the pelvic cavity before and after 2 to 4 cycles of pembrolizumab therapy. (A) A lesion was found in the posterolateral right psoas muscle. (B) The lesion became larger after 2 cycles of treatment. (C) Lesion size was reduced significantly after 3 cycles of treatment. (D) Lesion size was further reduced after 4 cycles of treatment.

**Figure 3:**
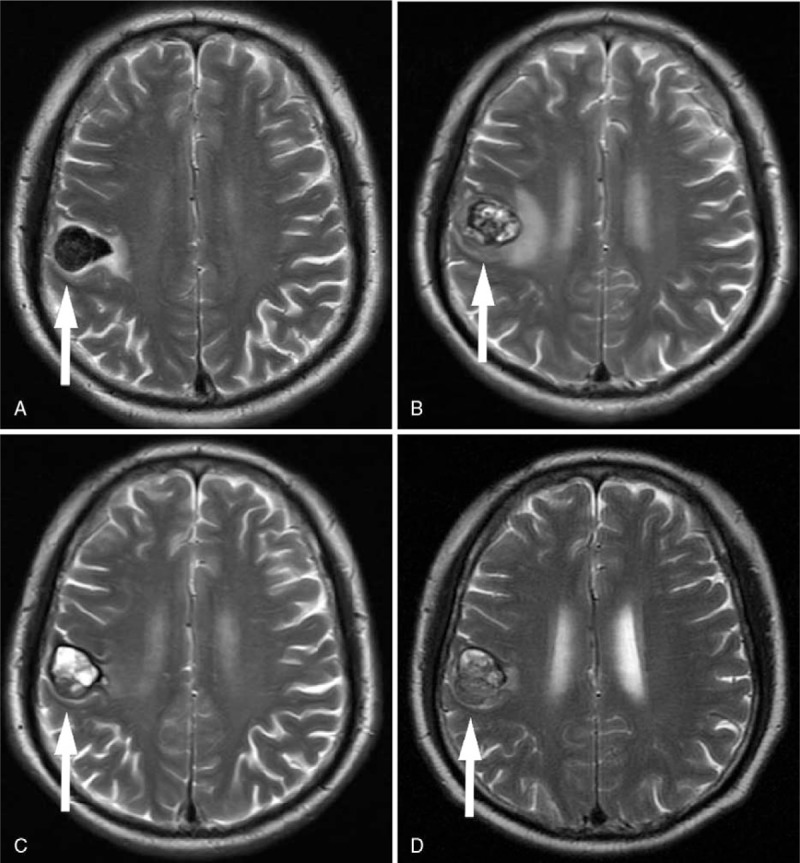
Enhanced MRI of the brain before and after 2 to 4 cycles of pembrolizumab therapy. (A) A lesion (2.4 cm × 1.9 cm) with low signal intensity on T2-weighted sequences was present in the right posterior frontal lobe. (B) The lesion (2.5 cm × 2.1 cm) showed mixed signal intensity with obvious surrounding edema after 2 treatment cycles. (C) The edema disappeared and the lesion was smaller (2.2 cm × 1.8 cm) with signal intensity increased after 3 cycles of treatment. (D) The signal became even and glial hyperplasia was found in the lesion (2.2 cm × 1.6 cm) after 4 cycles of treatment.

**Figure 4:**
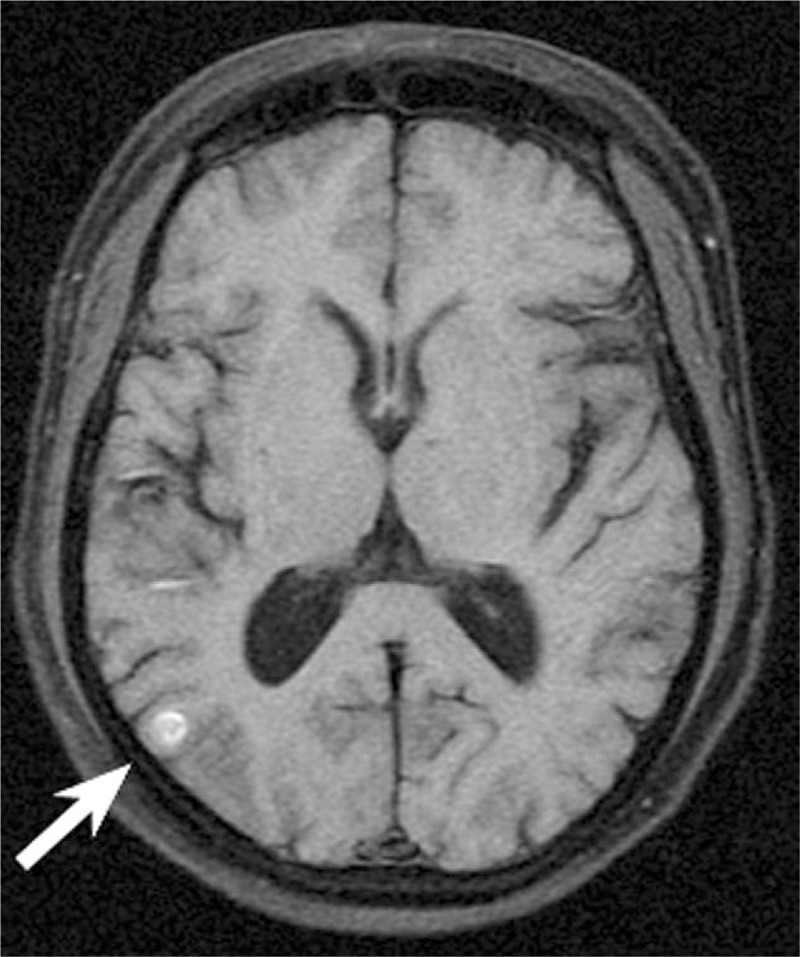
Enhanced MRI showed a new lesion in the right occipital lobe after 4 cycles of pembrolizumab therapy.

In January 2016, the patient presented headache, dyspnea, and cough. CT showed discrete scattered lesions in both lungs and the pelvic cavity (Figs. 1A and 2A). MRI revealed a mass measuring 2.4 × 1.9 cm in the right posterior frontal lobe (Fig. 3A). The patient commenced therapy with 6 cycles of the PD-1 inhibitor Pembrolizumab (2 mg/kg every 3 weeks). He experienced immunorelated adverse events in April 2016 (during the second cycle), we gave him cortisol to treat immunorelated disease until his pneumonia was founded (Table 1). The symptoms were relieved after 1 cycle, but the lesions continued to grow. After 3 cycles, the number and size of lesions in the lung, abdomen, and pelvic cavity had decreased markedly (Figs. 1C and 2C). The intracranial lesion presented clear margins, localization, the disappearance of edema, and glial hyperplasia (Fig. 3C and D). After an additional cycle, a new lesion appeared in the right occipital lobe (Fig. 4), with high signal intensity on T1- and T2-weighted images, and the other lesions remained stable. We recommended a biopsy of the new lesion, but the patient refused due to the invasiveness of the examination. Another 2 additional cycles of Pembrolizumab, the treatment was stopped due to the patient's inability to pay for it and a decline in his performance status. He then received palliative treatment at a local hospital and died within a short period due to severe intrapulmonary infection. The OS time of this patient was 7 months after metastasis.

**Table 1:**
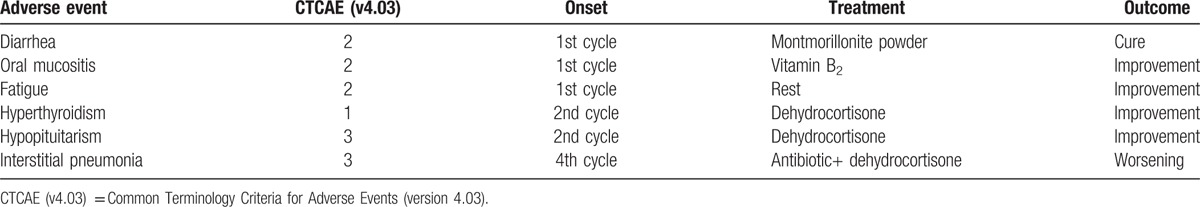
Immunorelated adverse events.

## 3 Discussion

Many drugs (e.g., fotemustine and temozolomide) are not effective for the treatment of melanoma with brain metastasis due to the blood–brain barrier.^[7]^ With emerging studies of immune checkpoint inhibitors (anti-CTLA-4/anti-PD1), immunotherapy has brought hope to MBM patients. Clinical trials have proven the efficacy of ipilimumab (the CTLA-4 inhibitor), with reported central nervous system control rates of 16–65% and median OS times of 2.5 to 29.3 months, grade 3 to 4 toxicity was 6 to 44.7% (Table 2).^[8–20]^ However, few trials have specifically studied the activity of anti-PD1 drugs in MBM patients. Some researchers have explored the efficacy of Pembrolizumab in MBM patients, the outcome seemed optimistic combined with radiotherapy (Table 2).^[21–24]^ However, these studies had many limitations, including the incompleteness of survival data, small samples, retrospective nature, and exclusion of patients with symptomatic or large brain metastases. Hence, a carefully designed prospective large scale clinical trial is urgently needed.

**Table 2:**
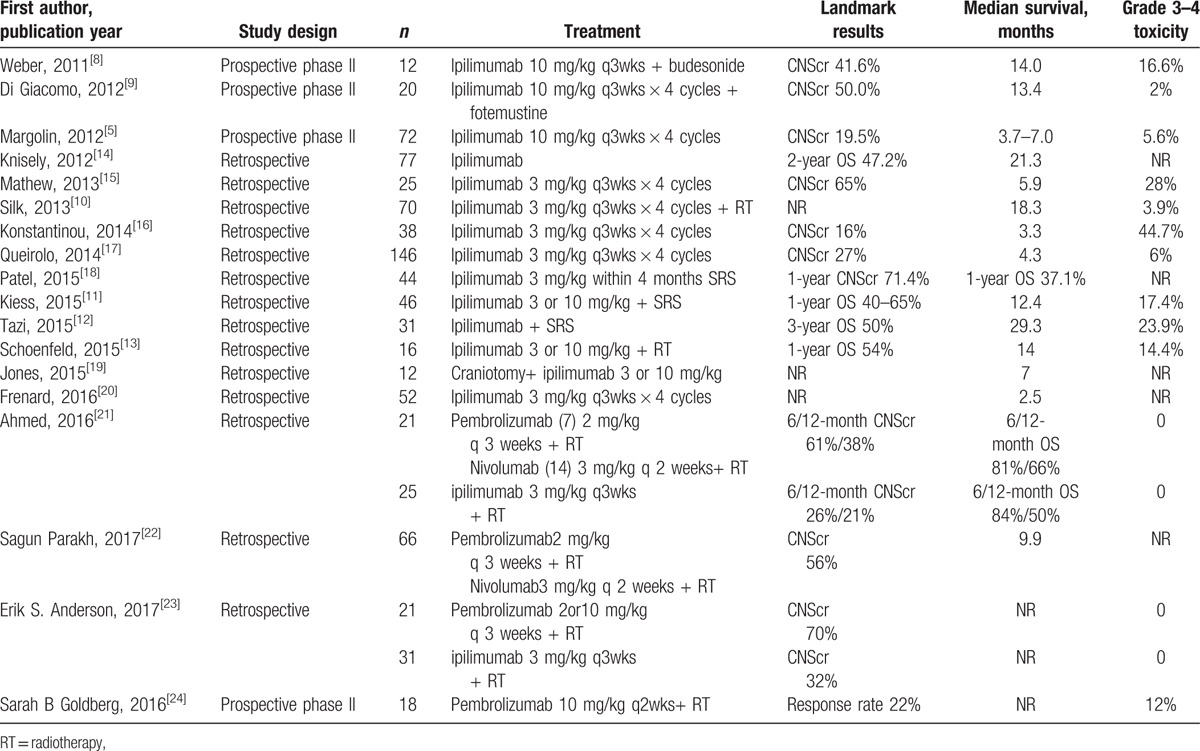
Studies of immunotherapy in patients with melanoma and brain metastases.

The mechanism of immune checkpoint inhibitors in patients with MBM is not clear. Previous findings suggested that these drugs could not penetrate the blood–brain barrier.^[25]^ Margolin et al^[5]^ argued that these drugs worked on the general immune system, and activated the T cells that might cross an intact blood brain barrier. Silk et al^[10]^ considered that the blood brain barrier was damaged in contrast enhancing MBM. Consequently, immune checkpoint inhibitors could be able to enter and activate T cells. An important issue currently is to find the predictive and prognostic factors of MBM patients. One study of stage III reported that loss of expression of phosphatase and tensin homolog deleted on chromosome ten (PTEN) will lead to activation of the phosphatidylinositol 3 kinase/protein kinase B (PI3K-AKT) pathway, and promote MBM.^[26]^ Activation of Janus protein tyrosine kinase signal transducer and activator of transcription (JAK-STAT) signaling pathway and factors like vascular endothelial growth factor A (VEGF-A), heat shock protein (HSPE), connexins, were also risks of MBM though enhancing blood brain barrier (BBB) hyperpermeability or invasiveness of melanoma cells to the brain.^[27]^ The high density of tumor infiltrating lymphocytes, a neo-antigen signature and high mutational load were associated with the survival of MBM patients.^[28]^ Patrick detected a strong negative correlation of programmed death ligand 1 (PD-L1) with brain metastasis size, with a higher PD-L1 level in brain metastases smaller than 30 mm in diameter.^[29]^ Recently, several studies described that patients with PD-L1 expression have a better objective response rates and longer OS than those with PD-L1 negativity in a variety of tumors,^[30,31]^ but not in others.^[32,33]^ PD-L1 cannot be used as a prognostic factor for the following main reasons: 1) biopsy at a single point fully encompassing PD-L1 expression is difficult, as the dynamic interaction between immunity and the neoplasm (PD-1/PD-L1 axis) is considered to form part of the adaptive immune system^[30]^; 2) the inflammatory cytokine IFN-γ can unregulated PD-L1 expression (biopsies taken during early anti-PD-1 treatment showed increased expression in previously PD-L1 negative tumors)^[34]^; 3) no standard method for PD-L1 detection and precise definition of its positive expression have been established^[35]^; and 4) clinical trials have demonstrated substantial response to anti-PD-1 drugs in a significant number of patients with PD-L1-negative tumors^[36,37]^ and the use of PD-L1 positivity as a biological marker will eliminate the potential benefit to these patients. Therefore, the presence of PD-L1 should not be a unique factor governing the administration of anti-PD-1 therapy.

Approximately 50% of melanoma patients have BRAF mutation and V600E point mutation is the most frequently observed. BRAF tyrosine kinase inhibitors like Vemurafenib and Dabrafenib have shown improved disease control rates of up to 92% in V600E mutation patients, while, the response of BRAF inhibitors is merely 6 to 20% in V600K mutation patients.^[38]^ Unfortunately, a disease will recur within 12 months in almost all patients treated with BRAF inhibitors; they developed resistance to BRAF inhibitors most likely trough mutations resulting in mitogen-activated protein kinase (MAPK) reactivation.^[39,40]^ Studies had demonstrated that combination of BRAF and MEK inhibition will prolong progression free survival in extracranial metastatic melanoma; however, there is still no definitive conclusion in MBM patients. Preclinical data suggested a better brain penetration of Dabrafenib.^[41]^ According to National Comprehensive Cancer Network (NCCN) guidelines, genetic testing should have been done on the dissected lymphoma node to get BRAF V600E status as Vemurafenib is the first line therapy for metastatic melanoma. Due to our hospital was unable to conduct the V600E mutation and Dabrafenib/Vemurafenib was not available in China, we did not consider the target therapy in our case.

Melanoma is relatively radiation resistant, median survival was 3.4 months and 2.1 months, respectively, for MBM patients who underwent WBRT and supportive care; the intracranial metastatic control and OS following WBRT+SRS were not different than after SRS alone.^[27]^ Current recommendations are for use of SRS for patients with ≤4 brain metastases that ≤3 cm in diameter.^[27]^ There are many lesions in the lungs and the pelvic cavity (Figs. 1A and 2A) when the brain lesion was found, as well as his performance status was poor, we considered that he could not tolerate and benefit from the surgery or radio surgery, so we recommended immunotherapy. Studies have demonstrated enhanced efficacy of anti-PD-1 antibodies when combined with radiotherapy (Table 2). In theory, antigen release due to cell death caused by the radiation increases the T cell mediated immune response facilitated by the immune checkpoint inhibitor treatment. Radiation enhances antitumor immunity by activation of dendritic cells and up-regulation of adhesion molecules, such as intercellular cell adhesion molecule (ICAM) and vascular cell adhesion molecule (VCAM) in the BBB. Effector T cells activated by anti-PD-1treatment have high expression of adhesion molecules ligands, such as integrins, which would increase their penetration of BBB while providing for a re-activation of the T cells already present at the tumor site.^[42]^ However, the best combined strategy is still unknown. In a retrospective study, they found that immune checkpoint therapy administered within 4 weeks of SRS (either prior to or after SRS) results in favorable OS and intracranial disease control compared to if immunotherapy and SRS are used more than 4 weeks apart.^[43]^ What would be happened if we add the doses of anti-PD1 drugs? Clinical trials have shown no improvement in the objective response rate and no difference in adverse effects of 10 mg/kg relative to 2 mg/kg Pembrolizumab in 3-weekly intervals in patients with melanoma.^[44–46]^ Whether the results would be similar in patients with brain metastases remains unknown. Other combined treatments (i.e., with 2 checkpoint inhibitors or one checkpoint inhibitor with chemotherapy/ targeted therapy) have also been explored, a better response was achieved.^[28]^ Despite initial success, there are still some challenges including patient selection and predictors of response, drug tolerance, optimizing combination strategies and control of adverse effects.^[30]^ More clinical trials about the survival benefit of combination regimes specially concentrating on brain metastatic are therefore required.

Pneumonitis has been reported in 0.4 to 4.3% of patients receiving Pembrolizumab at a median time of 5 months after anti-PD-1 therapy^[47]^; our patient developed severe pneumonitis 3 months earlier. The incidence rates of hypophysitis and hypothyroidism were 0.5% and 1.2%, respectively, at a median time of 1.3 to 1.7 months after anti-PD-1 drug used.^[47,48]^ Our patient developed hypophysitis and hypothyroidism at 1.5 months, consistent with these reports. In clinical trials, the effects of immunotherapy seem to be influenced by steroids; for example, the remission rate was higher in patients who did not receive steroids, but whether this difference reflects antagonism between the drugs or the more severe condition of those using steroids remains unclear.^[17]^ Data from extracranial metastatic melanoma indicates that corticosteroid treatment for the control of immune related side effects does not alter response probability.^[49]^ Harter found that corticosteroids neither affect the amount of tumor-infiltrating lymphocytes (TILs) nor PD-L1 expression in MBM.^[29]^ Hence, we guess that steroid will not influence the effect of Pembrolizumab in MBM patients. We had used cortisol to treat immunorelated adverse events in our case, and found that the hyperthyroidism and hypopituitarism became better. Nevertheless, the patient developed severe pulmonary infection. Thus, the pros and cons of steroid use should be considered carefully for increasing the risk of infection, and the appropriate dose and duration will be determined in future studies.

Immune checkpoint inhibitors activate T cell and result in subsequent disease control, some patients present initially with a stabilization of the disease and delayed tumor shrinkage only after several weeks or months of treatment.^[28]^ The lesions observed in our case became smaller after 3 cycles of Pembrolizumab reflecting the delayed nature of immunotherapy's anti-tumor effects. Patients with pulmonary metastases have shown good responses to immune checkpoint inhibitors,^[50]^ and we observed a better response in our patient's pulmonary lesions than in his other lesions, which may be related to the abundance of blood in the lungs. The antitumor effect is mediated by T cells and the initial influx of immune cell in the tumor tissue can cause pseudoprogression, which was most happened in the combination of immune checkpoint inhibitors and stereotactic radiosurgery.^[28]^ A new lesion appeared after 4 cycles of treatment in our patient, it might be pseudoprogression or inflammation rather than progression, to better determine the nature of this lesion, a biopsy was strongly recommended. We considered it progression, as it showed high signal intensity on T1- and T2-weighted images, unlike inflammation or pseudoprogression. However, the lesions in the lung and pelvic cavity became smaller. A mixed response was observed, with still stable extracranial metastases but progress of MBM. We speculated that this difference is related to the blood–brain barrier, or to the variable drug responses of different tissues.

The prognosis of brain metastasis melanoma patients was poor. The emergence of immune monitoring point inhibitors has brought hope to these patients. In the case reported here, a delayed and mixed response was observed after Pembrolizumab was used. Because of causing severe pulmonary infection, the use of steroids should be considered carefully when treating immunorelated adverse events. In addition, as data on tumor PD-L1 expression and OS are not consistent, the prognostic and predictive value of PD-L1 measurement is not clear. It seemed that the Pembrolizumab has a positive effect on MBM especially combined with other treatments. Despite initial success, many questions related to patient selection, predictors of response, drug tolerance, optimizing combination strategies, and control of adverse effects remain to be answered. With the development of numerous clinical trials and related questions to be resolved, we believe that more advanced and optimized immunotherapy benefitting patients with melanoma will become available.
